# Multiomics for Risk Stratification in Atherosclerotic Cardiovascular Disease

**DOI:** 10.1161/CIRCGEN.125.005451

**Published:** 2026-03-25

**Authors:** Liv Tybjærg Nordestgaard, Paolo Magni, Miron Sopić, Melody Chemaly, Ljubica Matic, Nawar Dalila, Fábio Trindade, Brooke N. Wolford, Zulema Rodriguez-Hernandez, Núria Amigó, Alberico L. Catapano, Lluìs Masana, Yvan Devaux

**Affiliations:** 1 Department of Clinical Biochemistry, Copenhagen University Hospital-Bispebjerg and Frederiksberg, Denmark (L.T.N.).; 2 Medical Research Council Integrative Epidemiology Unit, Population Health Sciences, University of Bristol, United Kingdom (L.T.N.).; 3 Istituto di Ricovero e Cura a Carattere Scientifico (IRCCS) MultiMedica, Milan, Italy (P.M., A.L.C.).; 4 Department of Pharmacological and Biomolecular Sciences, Università degli Studi di Milano, Italy (P.M., A.L.C.).; 5 Faculty of Pharmacy, Department of Medical Biochemistry, University of Belgrade, Serbia (M.S.).; 6 Division of Vascular Surgery, Department of Molecular Medicine and Surgery, and Center for Molecular Medicine, Karolinska Institutet and Karolinska University Hospital, Stockholm, Sweden (M.C., L. Matic).; 7 Department of Clinical Biochemistry, Copenhagen University Hospital-Rigshospitalet, Denmark (N.D.).; 8 RISE-Health, Department of Surgery and Physiology, Faculty of Medicine, University of Porto, Alameda Prof. Hernani Monteiro, Porto, Portugal (F.T.).; 9 HUNT Center for Molecular and Clinical Epidemiology, Department of Public Health and Nursing, Norwegian University of Science and Technology, Trondheim, Norway (B.N.W.).; 10 Department of Data Driven Medicine, Institute of Epidemiology and Prevention, Faculty of Medicine and Medical Center, University of Freiburg, Freiburg, Germany (Z.R.-H.).; 11 Biosfer Teslab, Reus, Spain (N.A.).; 12 CIBER of Diabetes and Associated Metabolic Disease (CIBERDEM), Instituto de Salud Carlos III, Madrid, Spain (N.A.).; 13 Department of the Vascular Medicine and Metabolism Unit, Lipid RDI Cluster, University Hospital Sant Joan, IISPV, CIBERDEM, Universitat Rovira i Virgili, Reus, Spain (L. Masana).; 14 Cardiovascular Research Unit, Department of Precision Health, Luxembourg Institute of Health, Strassen (Y.D.).

**Keywords:** atherosclerosis, cardiovascular diseases, epigenomics, epitranscriptomics, genomics, metabolomics, proteomics

## Abstract

Atherosclerotic cardiovascular disease (ASCVD) remains a leading cause of morbidity and mortality worldwide. Preventing ASCVD is of utmost importance; however, a large proportion of preventable cases is not discovered early enough to initiate relevant treatment. Risk stratification for ASCVD includes classical risk factors, such as sex, age, smoking habits, blood pressure, cholesterol levels, and diabetes. Current risk prediction models, including the Systematic Coronary Risk Evaluation 2 algorithms, are designed for individuals aged 40 to 69 years and relate to 10-year risk and not to lifetime risk, thereby being inaccurate for the young. Another problem is the underdiagnosis of events in women, thereby underestimating risk. Multiomics, encompassing genomics, epigenomics, transcriptomics, epitranscriptomics, proteomics, and metabolomics, offers new opportunities. Polygenic risk scores derived from genomic data may improve ASCVD risk classification. While genomic risk is established at inception, epigenomics captures the influence of environmental exposures over the lifespan through dynamic DNA modifications that regulate gene expression. Proteomics-based prediction reflects interactions between genetic inheritance, and modifiable and nonmodifiable influences. Transcriptomic analyses of carotid plaques have clustered human atherosclerotic lesions into distinct molecular subgroups, and changes in RNA methylation of circulating blood cells have been linked to clinical outcomes after ASCVD. Metabolomics identifies metabolic signatures, including lipid subclass alterations, amino acid imbalances, and inflammatory markers, all associated with cardiovascular disease incidence. In this review, we highlight current challenges, explore potential solutions, and discuss how integrating multiple omic layers through computational modeling (multiomics) could enhance patient stratification, optimize clinical management, and reduce the global burden of ASCVD.

Atherosclerotic cardiovascular disease (ASCVD) remains a leading cause of morbidity and mortality globally, with devastating personal, social, and economic consequences.^1^ Preventing ASCVD is a priority for health care systems worldwide. While universal prevention strategies, such as public health campaigns promoting smoking cessation or increased physical activity, are critical, targeted strategies aimed at high-risk individuals are generally more efficient. This focus on high-risk populations is justified by the direct association between absolute cardiovascular risk and therapeutic efficacy. For example, a 22% reduction in relative cardiovascular risk achieved by lowering LDL-C (low-density lipoprotein-cholesterol) levels by 1 mmol/L has a markedly different absolute impact when applied to populations with high versus low baseline risk and is also dependent on the duration of treatment.^2^

The Framingham Study was the first research project designed to define risk factors for various diseases, including cardiovascular disease (CVD). The initial results were published in 1957, but it was not until 1998 that the first cardiovascular risk prediction formula was introduced.^3^ This formula identified sex, age, smoking habits, blood pressure, and cholesterol levels, with diabetes acting as a transversal enhancer, as the primary determinants of CVD. The risk was expressed as the percentage likelihood of experiencing a major cardiovascular event within 10 years. Following the Framingham study, numerous other prospective observational studies were conducted using similar methodologies across diverse populations.^4–8^

Currently, guidelines from the European Society of Cardiology and the European Atherosclerosis Society recommend the use of the Systematic Coronary Risk Evaluation (SCORE)-2 index. The SCORE-2 is derived from data involving 677 684 participants and 30 121 cardiovascular events across 45 cohorts in 13 countries.^9^ This large data set enabled robust statistical analysis. The original SCORE-2 formula is tailored for individuals aged 40 to 69 years, with a derivative model, SCORE-2-OP, developed for those aged 70 to 90 years^10^ and an adapted model for patients with diabetes (Systematic Coronary Risk Evaluation 2-Diabetes).^11^ Notably, the variables included in the SCORE-2 project are largely consistent with those identified in the original Framingham formula.

Despite the importance of identifying individuals at high risk, existing tools for individual-level cardiovascular risk prediction remain suboptimal. The concordance index (C-index), which evaluates the accuracy of predictive formulas by measuring their agreement with observed outcomes, ranges from 0 to 1. A C-index of 0.5 indicates no predictive ability (equivalent to random chance), while a C-index of 1 indicates perfect prediction. Clinical tools such as SCORE-2 generally show modest performance, with reported C-index values ranging from 0.67 to 0.81 depending on the studied population.^9^ Other prediction models, such as the FHS (Framingham Heart Study) hypertension model and the American Heart Association’s Predicting Risk of Cardiovascular Events, have been estimated to have a C-index of 0.84^12^ and 0.69 to 0.73,^13^ respectively. These limitations highlight the need for improved methods to identify individuals at increased risk of ASCVD, including improvement in translation from risk at a population level to that at the individual level. While a previous review from our network introduced the various omics approaches and their roles in ASCVD diagnosis and treatment,^14^ the aim of the current review article is to examine in greater depth how omics can inform ASCVD risk stratification. In addition to the previous review, we here provide a detailed overview of polygenic risk scores, proteomic models, and metabolomic approaches for risk stratification in ASCVD. Furthermore, we describe in detail the role of methylation-based disease risk scores, noncoding RNAs, microRNAs, circular RNAs, and RNA methylation profiles in ASCVD risk stratification.

## How Can Omics Improve Risk Stratification?

Predisposition to ASCVD, its onset and progression, until the occurrence of major cardiovascular events and death, follow a complex and often individual pathway within a long timeframe.^15^ Such complexity derives from the variable combination of modifiable and nonmodifiable, but possibly identifiable, factors. Therefore, any accurate assessment of ASCVD risk should rely on comprehensive approaches able to capture most contributing components to ASCVD itself. These considerations prompt a change of paradigm, shifting from traditional cardiovascular risk charts to novel personalized approaches. Indeed, the current paradigm for assessing individual ASCVD risk relies, as mentioned above, on clinical risk algorithms, such as the SCORE-2 system in Europe.^16^ However, SCORE-2 and other traditional risk charts have several weaknesses, as, for example, they do not apply to subjects below 40 years and do not include family history of ASCVD or other relevant parameters such as LPA (lipoprotein[a]). In addition, they underestimate the risk in women and subjects with obesity and have a 10-year perspective, which inevitably classifies younger people as low risk. However, whether a risk prediction model including those under 40 years of age would significantly improve risk prediction is unknown. Identifying the lifelong risk of a given individual is the challenge for the future, and analyzing global exposure to causal risk factors is a much needed approach.^17^

On the road to implementation of a more comprehensive and holistic approach to ASCVD assessment, extensive research has recently been conducted by combining data ranging from genotypes to phenotypes and including large sets of molecular intermediates that can be identified through the wide spectrum of omic disciplines.^18^ Several recent studies have shown that the integration of multiomic data can help unravel the complex pathophysiology of ASCVD and improve clinical prediction models.^19–21^ A recent example has been provided by the FHS through the combination of genome-wide association studies, DNA methylation (DNAm), and transcriptomic data by using multivariable regression models to investigate subclinical atherosclerosis and myocardial infarction (MI). This revealed novel important associations with genes linked to smoking, platelet function, and cardiac tissue homeostasis.^22^

The wealth of molecular data provided by different omic layers, including genomics, epigenomics, transcriptomics, epitranscriptomics, proteomics, metabolomics, and radiomics, when combined with traditional lifestyle and clinical data, offers great power for disentangling the complex interactions underlying atherosclerosis pathophysiology and ASCVD risk and occurrence. Due to the large size of data sets and their different nature, multiomics and systems biology need to use complex and dedicated mathematical modeling and specific approaches based on artificial intelligence (AI) and machine learning (ML) network analysis strategies to ultimately define robust predictive algorithms able to support ASCVD risk assessment, diagnosis, and, possibly, therapy.^18^

**Figure 1.**
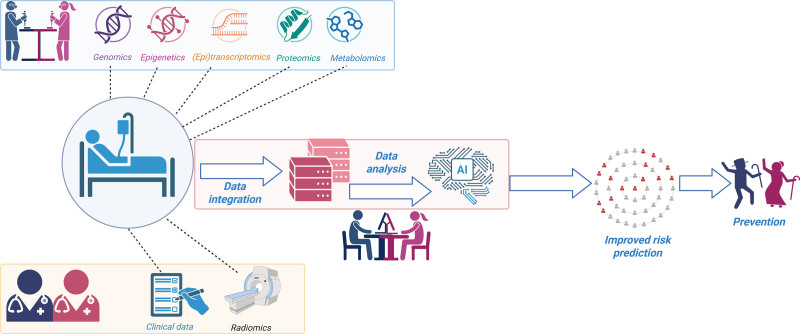
**Multiomics for improved atherosclerotic cardiovascular disease stratification.** Omics data in combination with clinical data and radiomics can be integrated and analyzed using machine learning and artificial intelligence to improve risk prediction in the general population. Created in BioRender.

The advantages offered by multiomics integration into clinical practice will be dependent on high-quality data sets, generated according to robust standard operating procedures, and the use of well-interpretable ML algorithms. These advancements would ultimately translate precision medicine from research into clinical care, especially in the cardiovascular and metabolic field, where risk stratification is pivotal to improve long-term outcomes. The aim of this review is to elucidate how different omics, separately and in combination, can help improve risk stratification in ASCVD See Figure 1.

## How Can Genomics Improve Risk Stratification?

### Opportunities

Genomics has been an important tool for risk stratification in ASCVD as individuals carrying specific genetic variants in the *LDLR*, *APOB*, and *PCSK9* (monogenic traits) have high levels of LDL-C and up to 13-fold increased risk of coronary heart disease (CHD).^23^ However, carriers of these variants are rare (prevalence=0.32% in the general population) and are only found in 6.7% of individuals with premature ischemic heart disease.^24^ However, with new techniques developing and larger databases available, the field of genomics is being transformed. Access to electronic health records in combination with exome-sequencing and whole genome sequencing has made possible improved population genomic screening and clinical management in patients with familiar hypercholesterolemia.^25^ Whole genome sequencing (which is the process of determining the entirety of the DNA sequence of an organism at a single time) has made possible the application of polygenic risk scores, including millions of genetic variants.^26^ Polygenic risk scores, which measure an individual’s inherited susceptibility to ASCVD across the entire genome, are an important emerging tool for risk prediction that adds additional information to clinical prediction models. A landmark study using the UK Biobank found that 8% of the population inherited a genetic predisposition to heart disease that conferred greater than 3-fold increased risk for CHD, which is equivalent to that conferred by (monogenic) mutations for familial hypercholesterolemia.^27^ Hence, focusing on the cumulative genetic risk rather than on rare genetic variation is likely to improve risk prediction for ASCVD.

The polygenic risk score is unique in that it is based on genetic variation, which is immutable from birth and, therefore, available early in life before other clinically used variables such as LDL-C are ever measured. However, it can also be considered a lifelong exposure, for example, genetically determined cholesterol levels are high across the life course in individuals at the highest tail of the polygenic risk score distribution. Thus, the cumulative risk of disease has notably different distributions at the top and bottom of the polygenic risk score distribution.^28^ In the UK Biobank, men in the highest quintile had a cumulative risk that increased exponentially around 40 years, reaching 10% cumulative risk by 61 years of age, whereas men in the bottom quintile did not reach 10% cumulative risk until 75 years of age. An age-specific effect of the polygenic risk score was observed in European-ancestry individuals across several diseases in multiple biobanks with a decreasing effect of polygenic risk score approximately linear with age.^29^ However, for CHD, the age-specific effect was significantly observed only in males. This is in line with previous results in a Norwegian population, which demonstrates the importance of age and sex interactions for optimally identifying individuals at risk of coronary artery disease (CAD) due to their polygenic risk score.^29^ In a Finnish study, individuals in the top 2.5% of the polygenic risk score distribution had CHD onset 4.35 years earlier than those with an average polygenic risk score.^30^ Furthermore, 12.6% of individuals with early onset disease (<55 years) had a high polygenic risk score but not a high clinical risk. In comparison to traditional clinical risk scores, polygenic risk scores have performed better in some studies such as the UK Biobank study by Elliott et al,^31^ where the overall net reclassification improvement of CAD by the addition of a polygenic risk score to a clinical risk score was 4.0% (95% CI, 3.1%–4.9%; Table 2), and in the study by Vassy et al,^38^ where the net reclassification improvement for risk of CVD was 0.38 (95% CI, 0.07%–0.68%) for men and 6.79 (95% CI, 3.01%–10.58%) for women. Taken together, the evidence points to polygenic risk scores as useful predictors for ASCVD that can be used early in life for better risk stratification and disease prevention.

### Challenges

Although polygenic risk scores are promising tools for risk stratification in ASCVD, using genomics in ASCVD risk prediction also poses several challenges. First, it has not yet been robustly shown that genomics offers a superior alternative compared with traditional risk factors,^39^ and the effect of the genetic background on the traditional risk factors (eg, hypertension and dyslipidemia) might already be captured in a risk score using only traditional clinical risk factors.^39^ However, it could be argued that silent polygenic variants (mutations in DNA, which do not have an observable effect on the organism’s phenotype) would not be captured by traditional risk factors before more specific tests, such as an angiogram or an echocardiogram, are performed. Furthermore, to implement polygenic risk scores in clinical practice, a choice between the many different polygenic risk scores available must be made, and it is not yet clear which one performs the best (Table 1). The cost of performing polygenic risk scores, including millions of variants in the general population, is not yet feasible although a validated genetic score may be evaluated in each individual just once in the lifetime and, thus, might become economically sustainable. A general challenge in genetic research is that most studies and most available cohorts include only white Europeans, and this could be a problem when trying to extend the polygenic risk scores to individuals of other genetic ancestries.^39^ Furthermore, information on sex-specific effects is also lacking from most studies. Finally, a considerable challenge is choosing which parts of the population would benefit most from the use of polygenic risk scores. Arguably, those who would benefit most would be young individuals because they would have the most years to moderate a genetic risk discovered with a polygenic risk score. However, polygenic risk could also serve a purpose in secondary prevention or in high-risk individuals. In individuals with a family history of ASCVD, genomic testing is always warranted.

**Table 1.**
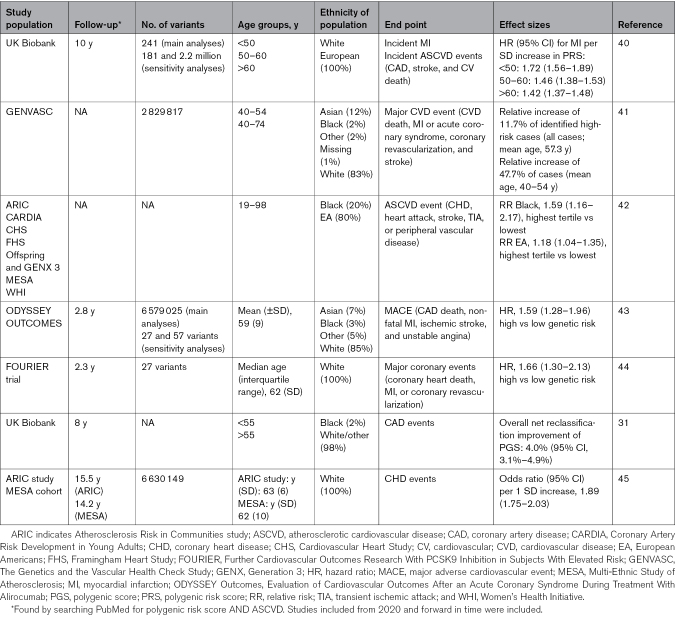
Examples of Polygenic Risk Scores

**Table 2.**
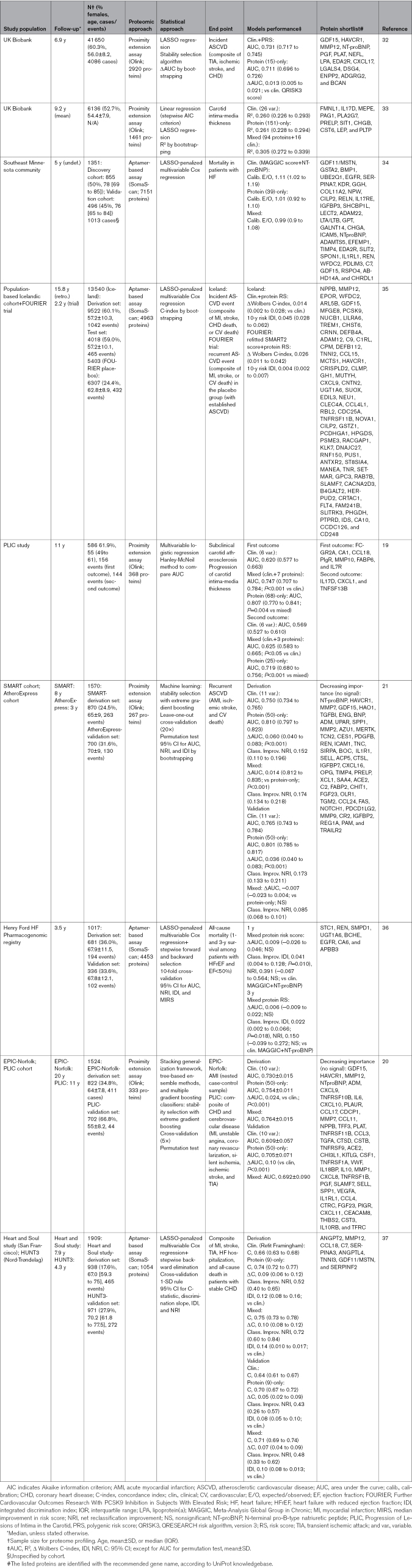
Examples of Proteomic Approaches for Risk Stratification in ASCVD

## How Can Epigenomics Improve Risk Stratification?

### Opportunities

Epigenomics is the study of epigenetic modifications of DNA. One advantage of epigenomics is that, unlike the DNA sequence, which is determined at fertilization, epigenetic changes occur throughout the lifetime of an organism, and thus, their assessment can capture the acquired impact of environmental factors on health and disease risk.^46^ Epigenomics studies how dynamic biochemical modifications regulate gene expression while preserving the DNA sequence. DNAm is one of the best understood epigenomic mechanisms involving the addition of methyl groups typically to the 5-carbon position of the cytosine base in a cytosine-phosphate-guanine dinucleotide sequence. In addition, DNAm is a key factor in genomic imprinting, cell differentiation, and X-chromosome inactivation.^47^

Over the past decade, multiple methylation-based disease risk scores have been developed to predict CVD risk.^48^ Methylation-based disease risk scores are calculated as weighted sums of an individual’s methylation beta values from a subset of cytosine-phosphate-guanine sites. Age-epigenetic clocks, based on the methylation-based disease risk score, have demonstrated that DNAm age mediates the association between CVD and cardiac age.^49,50^ Consequently, DNAm age could serve as an effective diagnostic tool to identify patient subgroups requiring more frequent clinical monitoring. DNAm can also serve as a proxy for protein levels through protein epigenetic scores. Epigenetic scores tend to have greater stability over time and offer stronger associations to disease outcomes than individual protein determinations, making them a useful tool for disease risk stratification. For instance, recent research has found that epigenetic scores for circulating protein levels are associated with CVD risk, regardless of traditional risk factors.^51^ In addition to DNAm, some studies have highlighted the importance of posttranslational modifications of histone^52^ and the posttranscriptional regulation of gene expression by noncoding RNAs^53^ on CVD.

### Challenges

The use of epigenomics for cardiovascular risk stratification encounters several challenges and limitations. First, epigenetic modifications are highly tissue-specific and cell-type–specific. Although DNAm studies account for the major blood cell-type composition,^54^ variations in cell subtypes cannot be excluded. In addition, DNA extracted from tissues (eg, heart and liver) derived from a mixture of cells adds complexity to the analyses. As an example, DNAm age may not always be the best indicator of cardiac aging, as highlighted by Mongelli et al,^49^ who discovered that the DNAm age of the heart is younger than that of blood. Second, the interindividual heterogeneity in epigenomic profiles, along with the epigenomics dynamic nature,^55^ can hinder the identification of universal long-term biomarkers for atherosclerotic-disease prediction. Third, CVD-epigenomic studies often focus on European populations and are, therefore, less generalizable to global populations, leading to biased risk models and exacerbated health inequalities. Finally, while epigenetic changes are associated with CVD, it is often unclear whether they are causal or merely a consequence of the disease process. This makes it difficult to use them for early diagnosis or risk prediction.

## How Can Transcriptomics and Epitranscriptomics Improve Risk Stratification?

### Opportunities

**Figure 2.**
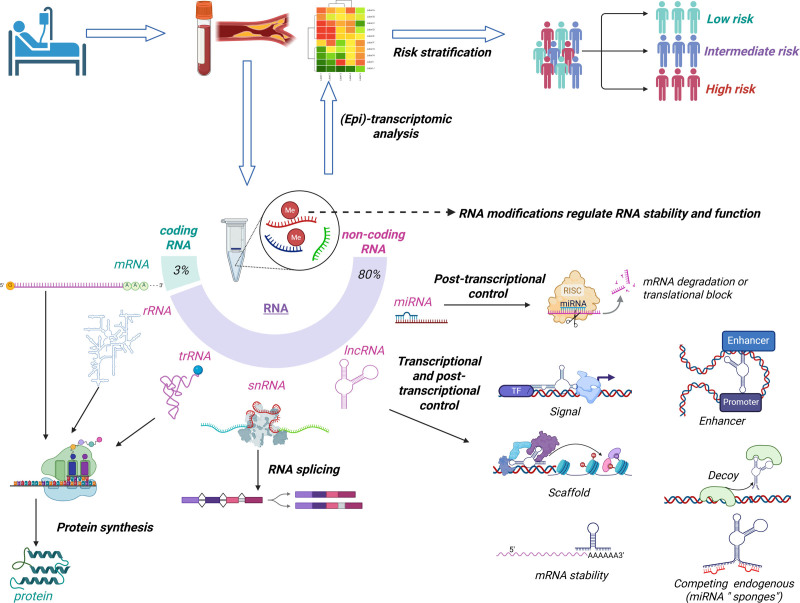
**The RNAs in atherosclerotic cardiovascular disease risk stratification.** Coding RNA (messenger RNA [mRNA]) makes up only ≈3% of the transcriptome, while noncoding RNAs comprise ≈80% and are essential for protein synthesis and regulatory functions. Ribosomal RNA (rRNA) and transfer-RNA (tRNA) are key to translation. Small nuclear RNA (snRNA) mediates RNA splicing. Long noncoding RNAs (lncRNAs) regulate gene expression by acting as signals, scaffolds, decoys, or enhancers. MicroRNAs (miRNAs) guide posttranscriptional silencing via mRNA degradation or translation inhibition. RNA modifications, such as methylation, influence RNA stability and function. Ultimately, (epi)transcriptomic profiling of atherosclerotic plaque and blood can uncover RNA signatures that enhance patient risk stratification into low-, intermediate-, or high-risk categories. Created in BioRender.

Transcriptomics is the study of the RNA molecules present in a cell or an extracellular compartment, such as the blood. Epitranscriptomics is the study of the mechanisms regulating RNA expression, stability, and function, such as splicing, editing, chemical modifications, and microRNAs. Contrary to protein-coding messenger RNAs, noncoding RNAs represent most RNA molecules and play important roles in gene regulation and ASCVD development (Figure 2). Present in body fluids, noncoding RNAs have shown interesting biomarker potential for CVD, particularly for risk stratification of ASCVD.^56^ Initial transcriptomic studies reported the capacity of easily detectable plasma microRNAs to stratify risk in patients after acute MI, toward a more personalized health care.^57^ Long noncoding RNAs, either linear or circular, are also detectable in the bloodstream, yet more steadily expressed in blood cells than in plasma or serum samples, and hold value as ASCVD biomarkers as well.^58^ Recently, peripheral blood mononuclear cell levels of circular RNAs HSPG2 (heparan sulfate proteoglycan 2) and YPEL2 (yippee-like 2) predicted atherosclerosis with an area under the receiver operating characteristic curve of 0.73.^59^ The applicability of circular RNAs for risk stratification capacity remains to be determined. With the advancement and increased availability of high-throughput sequencing techniques, more comprehensive transcriptomic approaches now provide deeper insights into the molecular complexity of atherosclerotic plaques. For example, bulk RNA sequencing of carotid plaques has enabled the clustering of human atherosclerotic lesions into distinct molecular subgroups, each characterized by different underlying biological pathways and clinical presentations.^60^ Such stratification based on transcriptomic profiles may improve patient risk assessment by identifying plaque phenotypes associated with higher vulnerability and adverse cardiovascular outcomes.

Changes in RNA methylation profiles of blood cells, such as that of the most widely present N6-methyladenosine, are associated with clinical outcomes after ASCVD^61^ although their risk stratification capacity needs to be demonstrated in large cohorts. The value of other RNA modifications, editing marks, and splicing patterns to risk-stratify ASCVD is currently under investigation.

### Challenges

While extensive research has been conducted to identify (epi)transcriptomics marks for risk stratification of ASCVD, translatability remains limited. Not because of technological constraints, because clinically applicable analytical platforms are now available, but possibly because of difficulties to generate robust and reproducible findings by research labs. This is due, at least partly, to poor standardization of experimental protocols.^60^ Therefore, the field needs initiatives to optimize the reproducibility of protocols to allow clinical application. Quality assurance tests where multiple laboratories analyze the same samples (Ring trials), for example, may be a useful tool for this.^62^

## How Can Proteomics Improve Risk Stratification?

### Opportunities

Given the demonstrated success of lipoproteins for risk stratification, the use of circulating proteins as surrogates is alluring in multifactorial conditions such as ASCVD. While traditional polygenic risk scores only account for inherited risk, proteome-derived risk prediction reflects the interaction between genetic inheritance and other nonmodifiable (eg, age and sex) and modifiable (eg, weight and diet) factors. The high-throughput analysis of thousands of proteins in targeted proteomic platforms, based on proximity extension (Olink) and aptamer-based (SomaScan) assays, measures many less-abundant disease-informative proteins, which are often masked in untargeted mass spectrometry workflows. The availability of this data, for example, through the UK Biobank Pharmacogenomics Project, has propelled the design of protein-centered models. These models show noninferior (or additive) performance to clinical-based ASCVD risk prediction ones for both primary^19,20,35,32^ and secondary prevention^32,34–37^ (Table 2). In most cases, adding protein variables or a protein risk score modestly, but significantly, improves the performance of clinical-based models. For instance, in a subset of 6136 patients from the UK Biobank with assessment of the carotid intima-media thickness by ultrasound, clinical (26 features) and protein-only (151 features) models isolated explained a similar degree of variance (R2≈0.26) in carotid intima-media thickness measures, but the combination of both (94 protein and 16 clinical features) rendered an R2 of 0.31.^33^ Given the lack of reproducibility in carotid intima-media thickness measures, a protein-based panel arises as a surrogate alternative to carotid intima-media thickness measurement, which is key for monitoring the risk of cerebrovascular disease.

A limitation of the proteomics-based risk stratification tools is the number of model features that hamper large-scale clinical utilization due to cost and time constraints. In this regard, using the least absolute shrinkage and selection operator or more advanced ML techniques is essential to narrow the selection to only the most stable features, facilitating model generalization.^32^ In a study aiming to predict mortality among patients with heart failure in the Southeast Minnesota community, over 7000 proteins were measured, from which 1300 proteins were associated with mortality (false discovery rate <1%). Using least absolute shrinkage and selection operator, the number was reduced to 38 proteins, resulting in an exceptionally calibrated model (expected/observed ratio, 1.01 [95% CI, 0.92–1.10] versus 1.11 [95% CI, 1.02–1.19]) in a model including the standard Meta-Analysis Global Group in Chronic Heart Failure risk score and NT-proBNP (N-terminal prohormone of brain natriuretic peptide).^34^ ML techniques help reduce dimensionality but also uncover nonlinear relationships between protein covariates that often remain hidden by traditional linear models. For instance, a 50-protein model built by stability selection with extreme gradient boosting improved the classification of patients with secondary ASCVD events (acute MI, ischemic stroke, and cardiovascular death) in a subset of the Second Manifestations of ARTerial diseases (SMART) cohort over an 11-variable clinical model (net reclassification index, 0.152 [95% CI, 0.110–0.196]) and was validated in the independent AtheroExpress cohort with a net reclassification index of 0.173 (95% CI, 0.133–0.211).^21^

A major advantage of assessing the proteome for stratification in patients with ASCVD is its ability to reflect temporal changes in risk, in stark contrast with genomic risk factors, which are immutable from birth (with the exception of clonal hematopoiesis). In a recent study, the proteomic predictor (15 protein-only model; area under the curve [AUC], 0.711 [95% CI, 0.696–0.726]) performed on par with the clinical risk predictor (QRESEARCH risk algorithm, version 3, clinical score; AUC, 0.702 [95% CI, 0.706–0.734]) and better than the genomics predictor (11 polygenic risk scores; AUC, 0.575 [95% CI, 0.558–0.591]) in predicting incident ASCVD among 40 000 individuals from the UK Biobank.^32^ For instance, a 9-protein risk score changed more than a refit Framingham score at the ≈11-year follow-up of stable patients with CHD of the Heart and Soul study (median annualized within-person change, 1.86% [95% CI, 1.15–2.54%] versus 1.00% [95% CI, 0.87–1.19%]).^37^ These observations underscore the need to define the optimal timeframe for protein-based risk prediction in clinical practice. For instance, in a nested case-control sample of the EPIC-Norfolk cohort, a 50-protein model outperformed (AUC, 0.754±0.011) a 10-clinical variables model (AUC, 0.730±0.015; ΔAUC, 0.024; *P*<0.001) in predicting MI over a median 20-year follow-up. However, Markov-chain Monte Carlo simulations identified 3 years as the optimal time horizon for predicting MI risk (protein model AUC, 0.803±0.093; clinical model AUC, 0.732±0.164^20^).

### Challenges

Despite the promise of proteomics for risk stratification, the studies conducted, thus far, have some limitations that must be overcome before clinical implementation. First, most studies have been done in homogenous populations, mainly consisting of individuals of European ancestry and the Northern Hemisphere. Efforts to include more diverse populations are needed to facilitate model generalization. In a study predicting mortality among patients with heart failure with reduced ejection fraction, 48% of the individuals included were Black, and the protein risk score improved patients’ classification of a combined MAGGIC-NT-proBNP model at 1 year.^36^ Second, proteomic studies have been restricted to targeted approaches using Olink and SomaScan platforms. While the number of assayable proteins is expected to increase, these methods have limited discovery potential compared with untargeted mass spectrometry, which might prevent the discovery of better-performing biomarkers. Furthermore, a proteomic score developed on one platform may not be used to calculate risk using measurements from another platform. Antibody and aptamer-based assays are also agnostic to posttranslational modifications, which can provide more specificity to prediction models. These platforms rely on relative quantification and show a bimodal distribution of correlations, hampering the definition of reference ranges required for clinical interpretation.^33^ Therefore, validation of protein risk models will necessarily encompass absolute quantification of the protein analytes. Finally, testing the response of proteome-derived risk stratification models to risk mitigation measures, such as lifestyle modifications, and medical and surgical interventions, will provide the final evidence of clinical utility. For now, available evidence shows that protein risk scores may modestly improve or attain similar performance to clinical-based ones, offering a complementary or alternative approach to standard risk stratification metrics.

## How Can Metabolomics Improve Risk Stratification?

### Opportunities

Metabolomics offers a promising approach to enhance cardiovascular risk stratification by capturing a detailed snapshot of an individual’s metabolic state. Beyond traditional risk factors, such as cholesterol levels, blood pressure, or smoking history, metabolomic profiling allows for the detection of subtle and rapid biochemical alterations that may precede overt clinical symptoms. This is particularly relevant for identifying residual risk in individuals who are already under standard preventive therapies but still experience cardiovascular events.^63^

Specific metabolic signatures, including alterations in lipid subclasses,^64^ altered amino acid concentrations,^65^ or inflammatory markers, have been linked to incident CVD, obesity,^66^ type 2 diabetes, and atherosclerosis.^67,68^ For example, advanced lipoprotein profiling through nuclear magnetic resonance can differentiate among proatherogenic lipoprotein profiles beyond cholesterol levels associated with reduced particle sizes and increased particle concentrations, providing insights into atherogenic dyslipidemia beyond what standard lipid panels offer (Table 3). This approach supports a more personalized risk assessment, which could lead to better-targeted interventions and monitoring strategies.

**Table 3.**
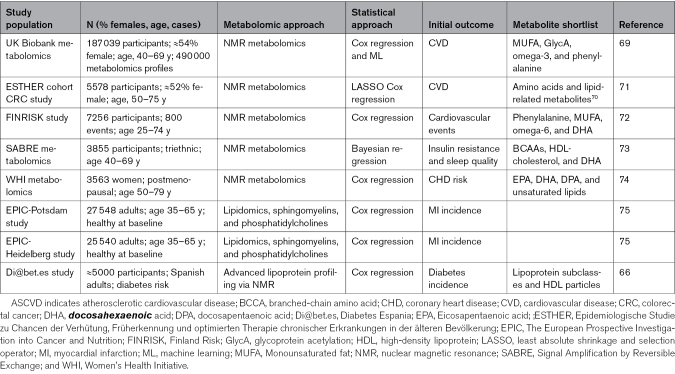
Examples of Metabolomic Approaches for Risk Stratification in ASCVD. Overview of Studies Including Different Metabolomic Approaches in ASCVD Risk Stratification

Moreover, metabolomics has the potential to uncover novel biomarkers that are mechanistically linked to pathophysiological pathways.^76^ By identifying metabolites involved in oxidative stress, endothelial dysfunction, or gut microbiota interactions, clinicians can not only improve risk prediction models but also gain insights into potential therapeutic targets.^77^ Integrating metabolomic data has already shown promise in refining predictive algorithms beyond traditional scoring systems, such as Systematic Coronary Risk Evaluation 2 or the Framingham Risk Score.^78^

### Challenges

The clinical translation of metabolomics into routine risk stratification faces several challenges. First, there is a need for standardization in sample collection, data acquisition, and analysis pipelines across laboratories.^79^ Variability in preanalytical and analytical conditions can significantly impact reproducibility and comparability of results. Second, large, diverse, and longitudinal cohorts are required to validate metabolomic markers and their added predictive value across different populations and clinical settings.^80^ Third, the integration of metabolomic data into clinical practice demands tools and workflows that are accessible to clinicians, as well as a clear demonstration of cost-effectiveness. While some platforms, such as nuclear magnetic resonance–based lipoprotein analysis, are already commercially available and partially integrated into clinical settings,^81,82^ the broader application of untargeted metabolomics remains limited by complexity, regulatory hurdles, and lack of reimbursement models. Fourth, the interpretation of metabolomic data is inherently complex. The high dimensionality of data and potential for overfitting necessitate robust statistical and bioinformatics approaches.^83^ Moreover, distinguishing between causal and associative biomarkers remains a major challenge, requiring careful study design and, ideally, integration with other omics layers (genomics, proteomics, and so on) and functional validation.^82^

Finally, a vast majority of metabolites have not yet been identified, and thus, the work on identifying and mapping all metabolites is still in its infancy.^84^ In summary, metabolomics holds great potential to refine cardiovascular risk stratification by providing a more nuanced, individualized assessment of disease susceptibility. Despite current methodological and translational challenges, ongoing technological advances and validation efforts are likely to pave the way for its broader clinical adoption.

## How Can Multiomics Improve Risk Stratification?

### Opportunities

The revolution in the fields of genomics, epigenomics, transcriptomics, epitranscriptomics, proteomics, and metabolomics has allowed the profiling of molecular and cellular pathways across multiple biological strata with never-before-seen resolution.^85–87^ However, to obtain a holistic understanding of the functional networks and interactomes underlining CVD, omics data integration is a must.^88^ This is key to achieving precision medicine, where a computational integration of omics data from a single patient can unlock a deep understanding of disease processes and open the possibilities to develop tailored targeted therapies for a broader group of patients. It is anticipated that multiomics data integration could bring us a step closer toward the concept of creating digital twins, a model for an entire biological system’s health trajectories, where personalized targets can be set to achieve the optimal health trajectory.^89^

Although a recent development, multiomics data integration has already led to some fascinating discoveries. Genomic and transcriptomic data integration resulted in the identification of 9p21 as the most significant locus in CAD^85^ and *SORT1* as a regulator of LDL-C and VLDL-C (very-low-density lipoprotein-cholesterol) levels.^86^ The identification of the 9p21 locus is a good example of the usefulness of multiomic methods in the understanding of a complex genetic locus where any one omic method likely would have failed. Integrating genomics with transcriptomics and proteomics within genome-wide association studies, such as the Framingham risk study, CARDIoGRAM (CAD Genome Wide Replication and Meta-Analysis)/CARDIoGRAMplusC4D (CAD Genome Wide Replication and Meta-Analysis Plus the CAD Genetics) studies, and the METASTROKE (Meta-Analysis of Genome-Wide Association Studies to Identify Genetic Risk Factors for Ischemic Stroke and Its Subtypes), allowed the discovery of single-nucleotide polymorphisms linked to expression quantitative trait loci and protein quantitative trait loci to causal CVD genes, such as *LMOD1*, *PECAM1*, *ATP1B1*, and *LPA*.^87^ In addition, genomics and metabolomics integration in a genome-wide association study led to the identification of loci in *ABO*, *NAT2*, *CPS1*, *NAT8*, *ALPL*, and *KLKB1*, which are associated with both metabolites and a high risk of CAD.^88^ Furthermore, integration of genomic, transcriptomic, and proteomic data highlighted major pathways related to mortality in patients with heart failure from the BIOSTAT-CHF study (The Biology Study to Tailored Treatment in Chronic Heart Failure), that is, a decreased activation of the cardioprotective *ERBB2* receptor, which can be modified by neuregulin.^90^ Recently, deconvolving bulk transcriptomic data using single-cell transcriptomics, along with a subsequent integration with genetics and clinical data from a large carotid atherosclerosis biobank, unraveled *ARNTL* as a modulator of smooth muscle cell population in plaques.^91^ Within the revolutionary field of radiomics applied to diagnostic computed tomography imaging, the integration of transcriptomic data with various quantified computed tomography image parameters predicted atherosclerotic plaque morphology and offered a deeper insight into the molecular signatures underlying radiomic plaque features.^92^ Together, these studies testify to the unprecedented power that multiomics integrations have already brought to the field of personalized cardiovascular medicine.

Multiple open-source databases are available, containing clinical data eventually to be combined with omics data. These databases represent a valuable source of information for data extraction and mining although this sometimes requires relevant bioinformatic expertise. In addition, the heterogeneity of the data contained in databases can represent a challenge, for example, for the homogenization of data coming from multiple studies or patient cohorts. Nevertheless, these databases provide bottomless reservoirs of data for reuse and generation of clinical models, according to the Findability, Accessibility, Interoperability, and Reusability principles.^93^ See Table 4 for an overview of available resources.

**Table 4.**
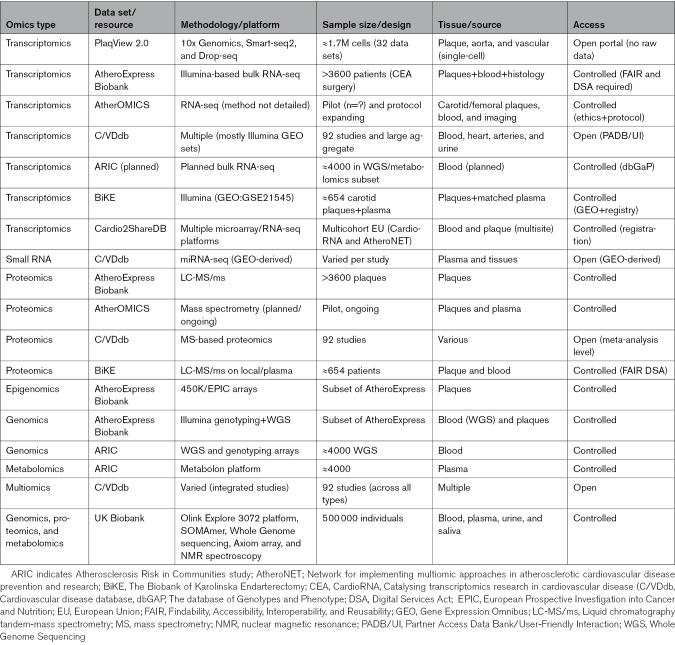
Multiomics Resources

Novel methods employing AI and ML techniques are answering the complex demands of this vast new field. Current computational methods for multiomics data integrations include supervised stratification (Data Integration Analysis for Biomarker Discovery Using Latent Variable Approaches for Omics Studies and Partial Least-Squares Discriminant Analysis), unsupervised stratification (Multi-Omics Factor Analysis and MixOmics), network-based approaches (Weighted Gene Co-Expression Network Analysis), or pathway/knowledge-based methods (based on Kyoto Encyclopedia of Genes and Genomes, for example). The basic principle is the same: multiomics data integration results in key molecules that are linked across data sets with a certain outcome or allow the most optimal subphenotyping of a cohort of interest.^94^ To this end, various research questions can be answered, where multiomics data integration can lead to, for example, a comprehensive molecular profiling of CVD, a deep profiling of the disease heterogeneity by identifying CVD disease subphenotypes (ie, endotypes), the discovery of biomarkers or therapeutic targets, new patient stratification methods for optimized clinical management, and more.^95^

### Challenges

To fully embrace the possibilities offered by multiomics data integration, several key hurdles need to be resolved. The heterogeneity of the data, their quality, and the diversity of the platforms used for profiling all render multiomics integration attempts challenging. Omics data are highly dimensional and present a skewed ratio of the number of samples compared with the number of measured biological features (a problem known as the curse of dimensionality).^96^ Another issue in multiomics data integration is the data missingness, which can, sometimes, be overcome by imputation methods.^97^ Multiomics data integration and analysis require large infrastructures supporting the amount of data while simultaneously facilitating data privacy, especially in projects running within large collaborative research networks and across several countries. Moreover, data visualization techniques need to be clear and simple, enabling actionable and condensed metrics for informed clinical applications.^95^ Finally, data reproducibility in multiomics integration is necessary and relies on the source code and training data being available.

Parallel with multiomics, multimodal data integration is also being developed, which combines molecular omics data with other modalities, such as diagnostic imaging, to use for clinical risk stratification in patients. Multimodal data integration typically applies AI/ML models and has the potential to synthesize even more complementary knowledge by summing individual measurements per patient, orthogonally from molecular to clinical scale. However, challenges are also present in multimodal data integration, including data scarcity, where patient information acquired during routine clinical care is not structured for research purposes or when data acquisition is collected differently across institutions (eg, sample collection and imaging equipment). Data infrastructures are another obstacle to multimodal data integration, necessitating laborious work for data harmonization before applying AI/ML approaches to health care research. These hurdles are currently the object of several large European Union projects, aiming to develop and implement federated learning approaches.^98^ With this, it is envisioned that multimodal data integration using AI/ML tools brings a unique, new promise to the clinic to truly advance individualized patient care. Interestingly, such ML algorithms may become more and more accurate as more data sets are implemented, thus showing a dynamic improvement capability, compared with the static nature of the currently used ASCVD risk scores.

## Comparison With Traditional Clinical Risk Scores

The advantage of genomics compared with traditional risk scores is that they can predict risk from an early age. Given that the effect of genetics on biomarkers that are also risk factors for ASCVD (such as cholesterolemia and blood pressure) will only become apparent later in life, genetics can be used much earlier and, thus, will be more efficient in the prevention of ASCVD.

Incorporating proteomics in multiomic studies will help capture the dynamic risk modified by age and modifiable risk factors, environment, changes in lifestyle, and medical interventions. Furthermore, considering the demonstration of a noninferior (or better) performance compared with clinical scores/models, proteomic screens may theoretically replace clinical risk models and minimize the need for clinical exams and interoperator variability. Although the field needs to mature, the inclusion of protein posttranslational modifications may also improve the specificity of the risk models. Finally, resorting to fully untargeted MS approaches, using the newer high-sensitivity and resolution platforms (eg, Trapped Ion Mobility Spectrometry With Time-of-Flight and Orbitrap Astral) is expected to broaden the discovery spectra and unveil new surrogate analytes that can improve current models.

Whether the gains in discriminatory power are sufficient to warrant incorporating multiomics into screening or prospective studies is still debated. For polygenic risk scores, increases in discriminatory power ranging from 0.4% to 7% have been reported,^30,31,38^ and arguably, only the latter represents an improvement substantial enough to justify the use of multiomics. Proteomic risk scores have been shown to match or exceed the performance of clinical risk models. In one study, a 50-protein model improved the AUC by 0.024 (*P*<0.001), with further gains when using a 3-year prediction horizon for MI.^20^ Another study reported a net reclassification index of 0.152 (95% CI, 0.110–0.196) for a similar 50-protein model.^21^ If a 5% increase in discriminatory power is taken as the threshold for implementing proteomics, only the latter result would meet this criterion. To date, there is no consensus on the discriminatory threshold justifying the inclusion of multiomics data in screening/prospective studies.

## Conclusions

Multiomic data will likely soon be able to help overcome some of the challenges currently faced in ASCVD risk stratification. Polygenic risk scores show promise although they have not yet been shown to substantially improve net reclassification of ASCVD events compared with classical clinical risk factors. Young individuals (eg, aged <40 years) are most likely to benefit from risk stratification by polygenic risk scores because they will have the most time to counteract any genetic risk with healthy lifestyle choices and preventative medication such as statins or *PCSK9* inhibitors.^39^ Epigenomics includes epigenomic markings from birth, as well as the impact of environmental factors throughout the life span. Although epigenomic risk scores, such as epigenetic scores, have been found to be associated with CVD risk, challenges for the implementation of epigenomics in the clinic remain, particularly the fact that epigenetic modifications are highly tissue-specific and cell-type–specific, and the fact that epigenetic changes are reversible. Transcriptomics analyses of carotid plaques have enabled the clustering of human atherosclerotic lesions into distinct molecular subgroups, and such stratification may improve patients’ risk assessment by identifying plaque phenotypes associated with higher vulnerability and adverse cardiovascular outcomes. However, it is important to note that to find a correlation with a biomarker to subclassify ASCVD in the future, additional data generation is necessary. Epitranscriptomic marks have shown regulation in ASCVD, yet have been poorly integrated in multiomic studies to date. This may change with the use of direct sequencing using nanotechnology that allows a combined assessment of transcript expression and modifications. Protein-centered models have shown comparable performance to clinical models for both primary and secondary prevention of ASCVD. The use of liquid biopsies as a source of information in proteomics is highly advantageous, as it enables continuous monitoring of risk and rapid assessment of adherence or response to medical interventions. However, a challenge to the use of proteomics-based risk stratification tools is the number of model features that hamper large-scale clinical utilization due to cost and time constraints. In addition, before clinical implementation, a paradigm shift from relative to absolute quantification will be necessary to definitively overcome interplatform differences (such as between Olink and SomaScan). Moreover, limited access to technology, the costs, and the need for highly trained personnel remain major barriers for implementation, making proteomics not the readiest omic for clinical application. Metabolomics has the potential to improve risk stratification in ASCVD by capturing a detailed snapshot of an individual’s metabolic state, including rapid alterations in lipid subclasses, which is particularly relevant for ASCVD. Multiomics data integration is critical for understanding the functional networks and interactomes underlining CVD and has already led to interesting discoveries such as the identification of 9p21 as the most significant locus in CAD.^99^

Challenges for multiomics data integration include the heterogeneity of data, their quality, the use of different analytical platforms, data missingness, and the need for high-performance computing. General challenges for the implementation of multiomics data in the clinic are the amount and complexity of data. For multiomics data to go from a purely experimental to a clinical application, the interpretation needs to be easily accessible for clinicians who are not omic experts. Furthermore, the price for multiomic data analyses is currently too high to be feasibly implemented in routine clinical examinations. Some omics data also come with important ethical considerations, especially genomics because of their potential for misuse in, for example, insurance cases. Furthermore, some of the data generation is not yet feasible. Although techniques especially for genomics and proteomics have been evolving, transcriptomics and epitranscriptomics are far from being clinically applicable methods.

### Perspectives

The future will likely bring multiomic data to risk prediction for ASCVD. Genomics is currently the modality closest to implementation although some proteins serve as long-recognized ASCVD biomarkers.^14^ These markers, however, were not identified through proteomic experiments, and proteomic approaches still carry substantial costs, which limit their cost-effectiveness. Metabolomics has the potential to contribute valuable information for clinical decision making, but when considering economic feasibility and comparability to traditional risk factors, it is still far from implementation in the clinic. Similar considerations apply to epigenomics, transcriptomics, and epitranscriptomics.^14^ This is especially likely for risk groups that are not captured by current prediction models, such as young individuals. However, the road to the general implementation of multiomic models in the clinic still includes many challenges that will have to be overcome before they can have a meaningful impact on ASCVD outcomes.

## ARTICLE INFORMATION

### Acknowledgments

This article is based on the work from COST Action AtheroNET, CA21153, supported by COST (European Cooperation in Science and Technology).

### Disclosures

Dr Devaux holds patents and licenses on RNA biomarkers of cardiovascular disease and is a member of the scientific advisory board of the molecular diagnostics company, Firalis SA. The other authors report no conflicts.
